# Ultra-wideband and Polarization-Insensitive Perfect Absorber Using Multilayer Metamaterials, Lumped Resistors, and Strong Coupling Effects

**DOI:** 10.1186/s11671-018-2810-0

**Published:** 2018-11-29

**Authors:** Si-Jia Li, Peng-Xin Wu, He-Xiu Xu, Yu-Long Zhou, Xiang-Yu Cao, Jiang-Feng Han, Chen Zhang, Huan-Huan Yang, Zhao Zhang

**Affiliations:** 1 Information and Navigation College, Air Force Engineering University, Xi’an, 710077 China; 2 Air and Missile Defense College, Air force Engineering University, Xi’an, 710051 China

**Keywords:** Metamaterial absorbers, Polarization, Subwavelength structures, Ultra-wideband

## Abstract

We theoretically and experimentally proposed a new structure of ultra-wideband and thin perfect metamaterial absorber loaded with lumped resistances. The thin absorber was composed of four dielectric layers, the metallic double split ring resonators (MDSRR) microstructures and a set of lumped resistors. The mechanism of the ultra-wideband absorption was analyzed and parametric study was also carried out to achieve ultra-wideband operation. The features of ultra-wideband, polarization-insensitivity, and angle-immune absorption were systematically characterized by the angular absorption spectrum, the near electric-field, the surface current distributions and dielectric and ohmic losses. Numerical results show that the proposed metamaterial absorber achieved perfect absorption with absorptivity larger than 80% at the normal incidences within 4.52~25.42 GHz (an absolute bandwidth of 20.9GHz), corresponding to a fractional bandwidth of 139.6%. For verification, a thin metamaterial absorber was implemented using the common printed circuit board method and then measured in a microwave anechoic chamber. Numerical and experimental results agreed well with each other and verified the desired polarization-insensitive ultra-wideband perfect absorption.

## Background

As an artificially engineered material, metamaterial has attracted significant interest because it exhibited fantastic electromagnetic properties unusual or difficult to obtain over the last decade [1–3]. With the rapid development, metamaterial with dynamical mass anisotropy has been applied to develop acoustic cloaks, hyperlenses, perfect absorbers, gradient index lenses [4–7], metalense, optofluidic barrier, polarization convertor, etc. [8–16]. In particular, the perfect metamaterial absorber (PMA) with ultrathin profile and near-unity absorption was firstly proposed by Landy et al. [6]. Relative to conventional absorbers, metamaterial absorber, which offers great benefits of thin profile, further miniaturization, increased effectiveness, and wider adaptability, has become promising applications of metamaterials. Later, researchers make several efforts on PMA to achieve wide incident angle absorption [17–19], multi-band absorption [20, 21], polarization-insensitive absorption [22–24], and the tunable absorption [25, 26]. However, absorbers with narrow bandwidth limit their applications in practice. Hence, it is necessary to design the ultra-broadband, polarization-insensitive, and thin metamaterial absorber.

To increase the absorption bandwidth, several methods such as by using the multi-resonance mechanism [27–38], the fractal structures [39], the multilayer [40–44], the magnetic medium [45, 46], and loading the lumped elements [47–49] have been proposed in the design of gigahertz and terahertz metamaterial absorbers. For instance, a broadband polarization-insensitive perfect absorber exhibiting a bandwidth of 9.25 GHz has been designed in a single layer based on the double octagonal-ring metamaterials and lumped resistances [50]. Additionally, a gigahertz perfect metamaterial-inspired absorber was proposed which was composed of three-layer substrates, double split-serration-rings, and a metal ground [51]. Although a relative bandwidth of 93.5% was obtained, the absorption bandwidth is still insufficient for applications, such as electromagnetic protection, stealth, and electronic warfare.

Different from the previous metamaterial absorbers, we proposed a thin and ultra-wideband perfect metamaterial absorber by combining the resonant and resistive absorptions using strong coupling effects. The absorber was composed of four dielectric layers, two metallic double split ring resonators (MDSRR) and several lumped resistors. The characteristics of polarization-insensitive and wide-incident absorption had been verified both numerically and experimentally. This perfect metamaterial absorber is promising for many practical applications such as radar cross scatter reduction, stealth, and electromagnetic protection in different flight platform.

## Methods

**Fig. 1:**
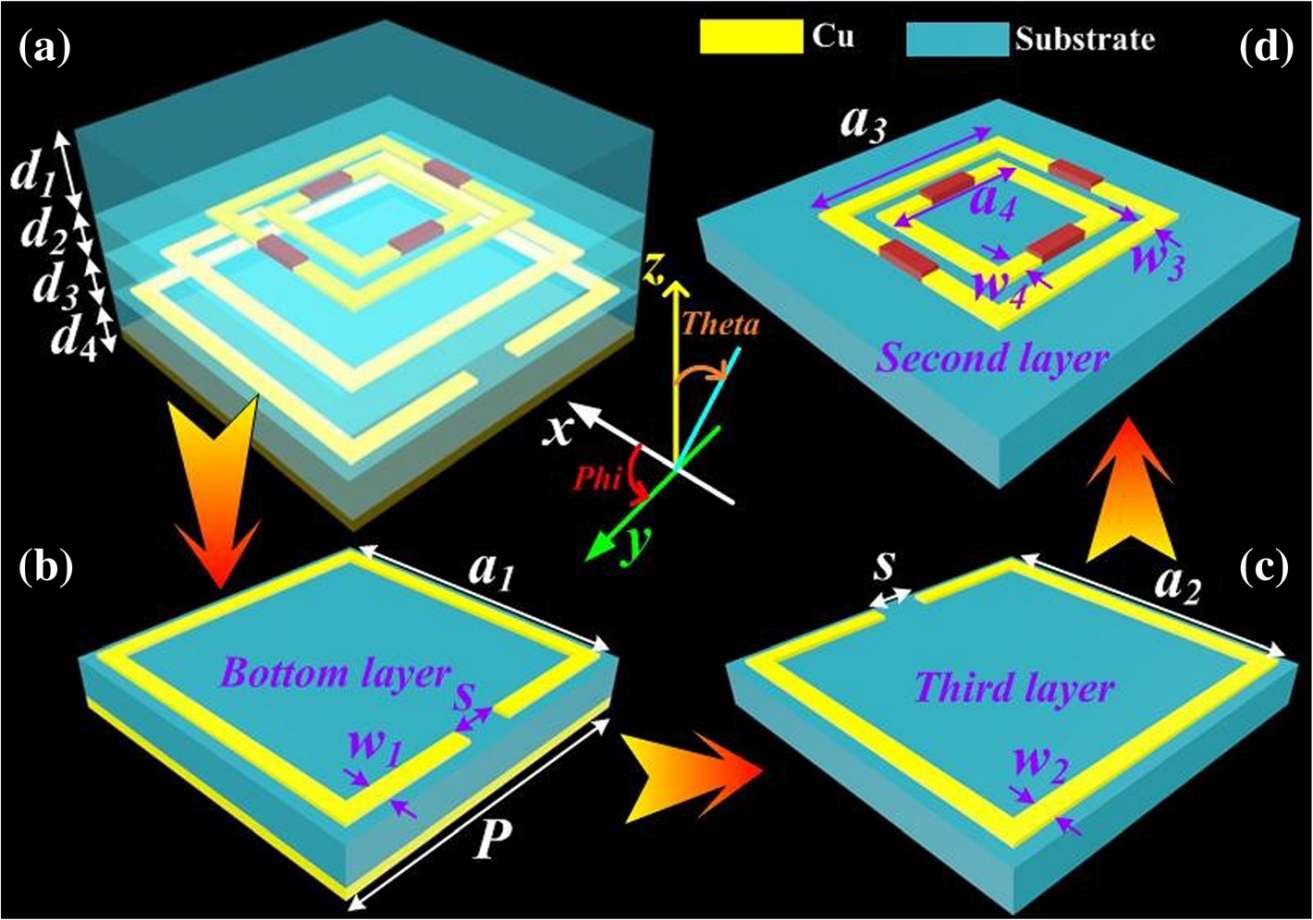
Schematic geometry of unit cell for the ultra-broadband perfect metamaterial absorber. (*a*) The 3D schematic of a unit cell. (*b*) The bottom layer of proposed PMA with the split ring resonator-II (SRR-II). (*c*) The third layer of proposed PMA with the split ring resonator-I (SRR-I). (*d*) The second layer of proposed PMA with first metallic DSRR (F-MDSRR) microstructure and four lumped resistances. The optimized parameters of the PMA were *d*_1_ = 2 mm, *d*_2_ = *d*_3_ = *d*_4_ = 1 mm, *w*_1_ = *w*_2_ = *w*_3_ = *w*_4_ = 0.8 mm, *P* = 8.4 mm, *R*_1,2_ = 60 Ω, *R*_3,4_ = 180 Ω, *a*_1_ = 7.8 mm, *a*_2_ = 6.6 mm, *a*_3_ = 5 mm, *a*_4_ = 3.4 mm, *s* = 1.2 mm. The thickness of the copper is 0.036 mm

The meta-atom of proposed ultra-wideband PMA consists of four dielectric layers, double metallic DSRR microstructures, and the lumped resistances in Fig. 1. To obtain the destructive interference, the top (first) dielectric spacer with a dielectric constant of 4.4 and a tangent loss angle of 0.02 is required as an antireflection coating substrate to enhance absorption bandwidth. The thicknesses of the four dielectric layer are *d*_1_, *d*_2_, *d*_3_, and *d*_4_. The dielectric constant and the tangent loss angle of the residual substrates are all 4.2 and 0.02 (ε_r_ = 4.2, tanδ = 0.02) respectively. As given in Fig. 1(d), the first MDSRR (F-MDSRR) microstructure with four lumped resistances is on the second substrate. The metallic split ring resonator-I (SRR-I) and split ring resonator-II (SRR-II) are respectively on the third and bottom substrate which make up the second metallic DSRR (S-MDSRR) microstructure. The F-MDSRR and S-MDSRR microstructures are copper with the conductivity of 5.8 × 10^7^S/m and thickness of 0.036 mm. The length of the meta-atom for the proposed PMA is *P* = 8.4 mm. As shown in Fig. 1 (b) and (c), the lengths of SRR-I and SRR-II are *a*_1_ and *a*_2_. Their widths are *w*_1_ and *w*_2_. The lengths and widths of F-MDSRR, as given in Fig. 1(d), are represented by *a*_3_, *a*_4_, *w*_3_, and *w*_4_. The resistances loaded on the inner and outer split rings are denoted by *R*_1,2_ and *R*_3,4_. And *s* denotes the length of the splits for F-MDSRR and S-MDSRR. The proposed PMA is designed, analyzed, and optimized in simulation. A full-wave electromagnetic simulation is performed by using the finite-element analysis-based ANSYS Electro-magnetics Suite 15.0. The proposed absorber is simulated and optimized with parameters of *d*_1_ = 2 mm, *d*_2_ = *d*_3_ = 1 mm, *d*_4_ = 1 mm, *w*_1_ = *w*_2_ = *w*_3_ = *w*_4_ = 0.8 mm, *P* = 8.4 mm, *R*_1,2_ = 60 Ω, *R*_3,4_ = 180 Ω, *a*_1_ = 7.8 mm, *a*_2_ = 6.6 mm, *a*_3_ = 5 mm, *a*_4_ = 3.4 mm, and *s* = 1.2 mm.

To explore the absorption mechanism for the proposed ultra-wideband PMA, the periodic boundary conditions (PBCs) and Floquet port were applied to simulate the infinite periodic cells. The electromagnetic (EM) wave would be gradually absorbed by the absorber according to the antireflection conditions. Both magnetic and electric resonances would be independently aroused, which could confine the wave into the PMA cell. The wave could be gradually absorbed by the dielectric loss. It could achieve that the magnetic permittivity equaled to the electric permittivity, resulting in the perfect absorptivity for incident EM waves. In more direct perspective, absorptivity was defined as [52–55]

1 $$ A(f)=1-T(f)-R(f)=1-{\left|{S}_{21}\right|}^2-{\left|{S}_{11}\right|}^2 $$

In order to maximize absorptivity *A*(*f*), we could minimize the transmission *T*(*f*) (*T*(*f*) *= |S*_21_*|*^2^) and the reflection *R*(*f*) (*R*(*f*) *= |S*_11_*|*^2^) simultaneously. The absorptivity could be calculated by *A*(*f*) = 1 − *R*(*f*) because the presented PMA was blocked by the metallic plate without patterns on the bottom layer (so the transmission was zero, *T*(*f*) *= |S*_21_*|*^2^ = 0). Hence, the absorptivity of the presented PMA could be calculated by

2 $$ A(f)=1-R(f)=1-{\left|{S}_{11}\right|}^2 $$

From the Eq.(2), it is obvious that the absorption is near to 100% (*A*(*f*) ≈ 100%) when the reflection is close to zero (*R*(*f*) ≈ 0). It is necessary to note that the S_11_ components include the reflection of co-polarized EM waves and the reflection of cross-polarized EM waves [56–58]. So the *S*_11_ components can be expressed as:

3 $$ {\left|{S}_{11}\right|}^2={\left|{S}_{11, xx}\right|}^2+{\left|{S}_{11, xy}\right|}^2 $$

Accordingly, based on the Eq.(3), the Eq.(2) could be evaluated by

4 $$ A(f)=1-R(f)=1-{\left|{S}_{11, xx}\right|}^2-{\left|{S}_{11, xy}\right|}^2 $$

where the *xx* and *xy* denote the co-polarization and cross-polarization. In the proposed PMA design, the ***|****S*_11_***|*** comprises the components of the co-polarization and the cross-polarization. Furthermore, the reflection of PMA at normal incidence is given by [6, 21]:

5 $$ R(f)=\frac{z_{\mathrm{eff}}(f)-{\eta}_0}{z_{\mathrm{eff}}(f)+{\eta}_0} $$

where *η*_0_, about 377 Ω, represents the free space impedance. *z*_*eff*_(*f*) is the effective impedance of PMA. The effective impedance includes the lumped resistances in proposed PMA, the surface impedance which is to obtain a large resonant dissipation and the substrate impedance due to the high tangent. By substitution of (5) in (4), the absorptivity *A* could also be written by:

6 $$ A(f)=\frac{2{\eta}_0}{\operatorname{Re}\left[{z}_{\mathrm{eff}}(f)\right]+i\cdot \operatorname{Im}\left[{z}_{\mathrm{eff}}(f)\right]+{\eta}_0} $$

where Re [*z*_eff_(*f*)] and Im [*z*_eff_(*f*)] are respectively the real part and the imaginary part of *z*_eff_(*f*). When the proposed PMA is at the resonant modes, the absorption is near to one (*A* = 1). From the expression of (6), we know that when *A* = 1, Re [*z*_*eff*_(*ω*)] and Im [*z*_*eff*_(*ω*)] can be calculated as:

7 $$ \operatorname{Re}\left({z}_{\mathrm{eff}}\left(\upomega \right)\right)=377\Omega, \kern0.5em \operatorname{Im}\left({z}_{eff}\left(\upomega \right)\right)=0 $$

It is found that the absorption is close to 100%, when the real part and imaginary part of the effective impedance are respectively close to 377 Ω and 0. The absorptivity is enhanced because of the different resonant modes. Generally, the excellent absorption could be obtained as the effective permittivity was equal to effective permeability. So the broadband absorption would be achieved by modulating the effective parameters.

The ultra-wideband metamaterial absorber was simulated by employing the commercial software, Ansoft High Frequency Structure Simulator (HFSS 18.0), which was based on the finite-element analysis method. In the calculation, a plane electromagnetic wave with the electric field along the direction of *x*-axis was used as the incidences, which was perpendicularly irradiated to the resonance structure along the direction of the *z*-axis (shown in Fig. 1). The frequency range from 1.0 to 30 GHz of the incidences had been used in simulation. The size of the incidences should be slightly larger than that of the presented period of the structure; at the same time, enough simulation times and the suitable boundaries (periodic boundaries in directions of *x*- and *y*-axis and perfectly matched layers in direction of *z*-axis) should be utilized for ensuring the accuracy of calculation results.

## Results and Discussion

**Fig. 2:**
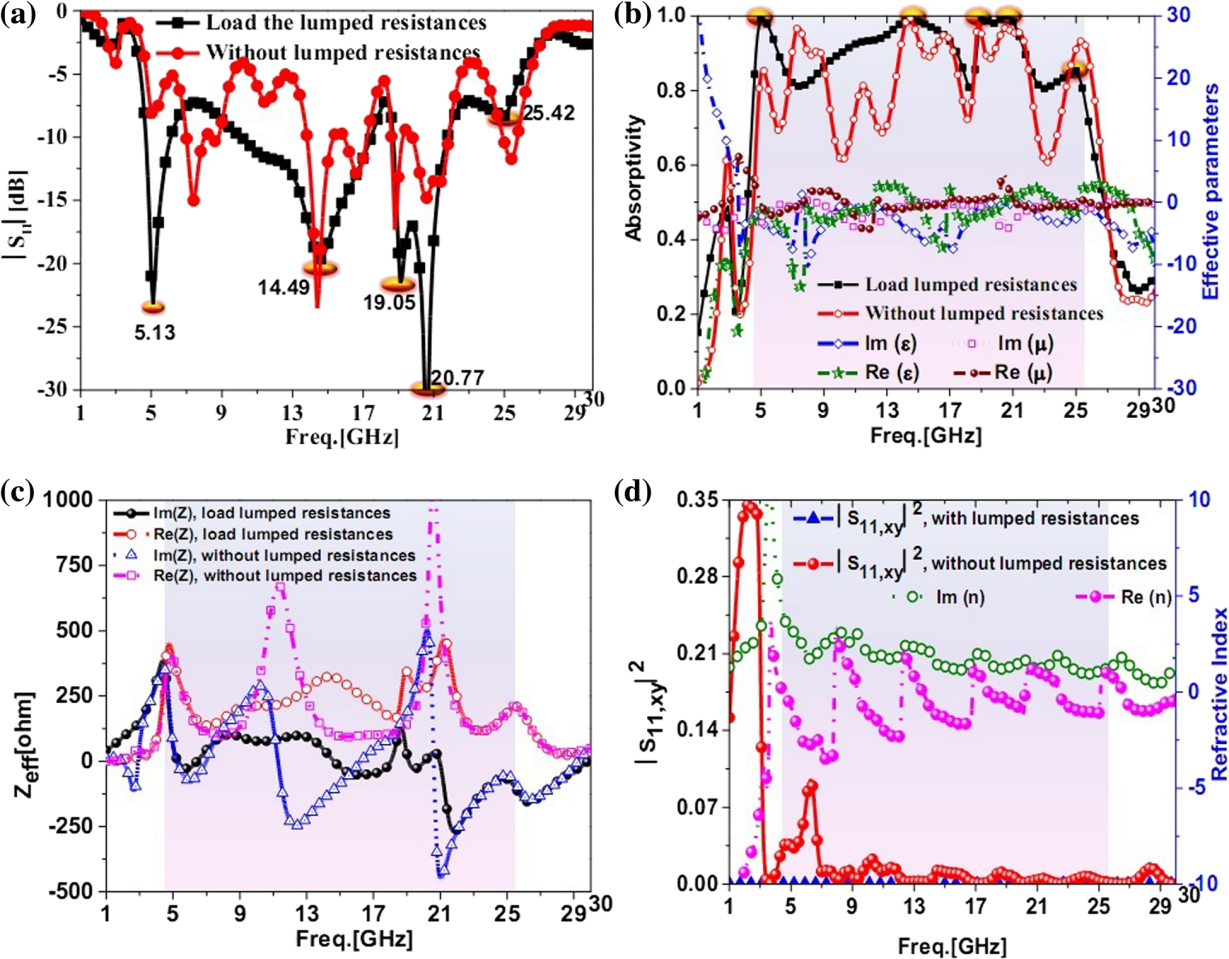
The simulated |S11|, absorption, effective parameters, effective impedances and refractive index from 1 to 30 GHz for the proposed ultra-wideband perfect metamaterial absorber loaded with lumped resistances and the same microstructure without the lumped resistances. **a** Simulated |S11| results. **b** Simulated absorption results and effective parameters. **c** The effective impedances of proposed PMA with lumped resistances and the same microstructure without lumped resistances. **d** The reflection components of the cross-polarization for the proposed PMA with lumped resistances and the same microstructure without lumped resistances and the refractive index of the presented PMA

The simulated amplitude of *S*_11_, absorption, effective impedance, and reflection components of the cross-polarization from 1 to 30 GHz are shown in Fig. 2. As shown in Fig. 2a, it can be seen that the proposed PMA exhibited ultra-broadband lower reflection from 4.5 to 25.5 GHz than that of the PMA using the same microstructure without lumped resistances. Especially, the differences between the microstructure with and without lumped resistances were evident from 9 to 14 GHz and from 19 to 21 GHz. In Fig. 2b, we could see that the ultra-broadband absorption from 4.52 to 25.42 GHz with absorptivity larger than 80% could be obtained for the proposed PMA and the absorption would deteriorate for proposed microstructure without lumped resistances obviously. The real and imaginary parts of effective impedance were respectively close to 377 Ω and 0 for the proposed PMA at the resonance frequency of 5.13, 14.49, 19.05, 20.77, and 25.42 GHz in Fig. 2c. The more the absorptivity near to 100%, the more the real and imaginary parts of effective impedance were respectively close to 377 Ω and 0. From Fig. 2d, the reflection components of cross-polarization were about zero for the proposed absorber from 1 to 30 GHz. It was necessary to note that the reflection components ***|****S*_11,*xy*_***|***^2^ of cross-polarization was about 0.35 at 2.8 GHz for the proposed microstructure without lumped resistances. This phenomenon was caused by the unsymmetrical structure and the weak resonator modes at the frequency. Therefore, the lumped resistances were important for the ultra-broadband PMA design. From Fig. 2b, d, the real part and imaginary part of the effective permittivity were respectively approximated to that of the effective permeability for the proposed PMA from 4.52 to 25.42 GHz. The imaginary part of refractive index was more than zero in this band. Consequently, the ultra-broadband can be exhibited for the presented PMA.

A parametric study was carried out by ANSYS HFSS Solver. In this study, it was main objective to achieve ultra-broadband absorption. According to this goal, some parameters of the lumped resistances *R*_1,2_ and *R*_3,4_ in the inner and outer split rings, the cell length *P* of the PMA, the length *s* of the splits for F-MDSRR and S-MDSRR, the thickness *d*_1_ of the antireflection coating substrate, and the thickness *d*_2_ were selected in the study.

**Fig. 3:**
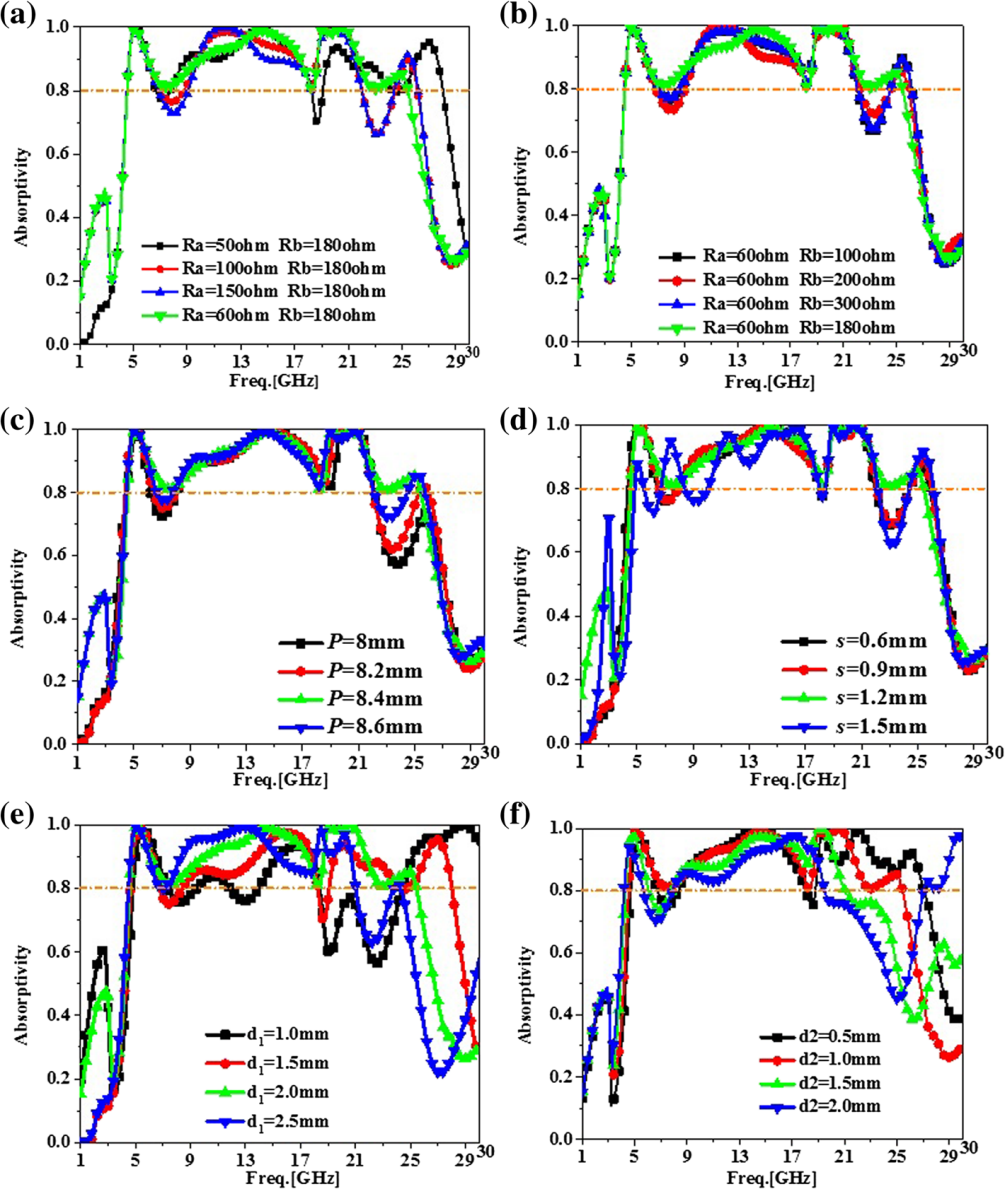
Absorption results from 1 to 30 GHz for the proposed ultra-wideband perfect metamaterial absorber with different parameters. **a** Absorption results of the PMA with different *R*_*a*_ values. **b** Absorption results of the PMA with different *R*_*b*_ values. **c** Absorption results of the PMA with different lengths of *P*. **d** Absorption results of the PMA with different lengths of *s*. **e** Absorption results of the PMA with different thicknesses of *d*_1_. **f** Absorption results of the PMA with different thicknesses of *d*_2_

Figure 3a shows the simulated absorption, when the proposed PMA adopted the lumped resistances of *R*_1,2_ = 50 Ω, 60 Ω, 100 Ω, 150 Ω. By adopting *R*_1,2_, the absorption was improved obviously from 19 to 25 GHz. While as *R*_1,2_ shifted from 50 to 150 Ω, the lumped resistances had slightly effect on absorption in low frequency. Hence, by selecting a proper value for *R*_1,2_ = 60 Ω, the proposed PMA obtained the ultra-broadband absorption. As shown in Fig. 3b, the *R*_3,4_ mainly affected the absorption in the range of 6~17 GHz and 21~23 GHz. For wideband absorption, *R*_3,4_ was chosen to be 180 Ω. The length was another critical parameter. The case with different lengths of PMA cell and splits in for F-MDSRR and S-MDSRR was studied. Figure 3c shows that the absorption from 21 to 25 GHz was very sensitive to the length *P* of PMA cell. To achieve wideband absorption, we selected *P* = 8.4 mm. In Fig. 3d, it was clear that the PMA had wideband absorption at low frequency and the bandwidth was influenced by *s* which was shifted from 0.6 to 1.5 mm. According to the standard of absorptivity more than 0.8, *s* = 1.2 mm was selected to obtain wideband absorption for the proposed PMA. The effects of the antireflection coating substrate thicknesses *d*_1_ are illustrated in Fig. 3e. It was obvious that the thickness *d*_1_ influenced the wideband absorption from 7 to 30 GHz and *d*_1_ = 2.0 mm was chosen for broadband PMA design. The absorption results with different *d*_2_ are given in Fig. 3f. It was clear that *d*_2_ was the key parameters for wideband PMA in high frequency. To achieve the ultra-broadband absorption, the optimized *d*_2_ of 1.0 mm was selected in PMA design.

From Figs. 2 and 3, it could be seen that the absorption bandwidth of the proposed PMA was sensitive to the thicknesses of *d*_1_ and *d*_2_, and the values of lumped resistances. Moreover, the splits in the F-MDSRR and S-MDSRR were necessary for achieving the wideband absorption in our design. Hence, the thicknesses and the lumped resistances needed to be optimized for ultra-broadband absorption.

**Fig. 4:**
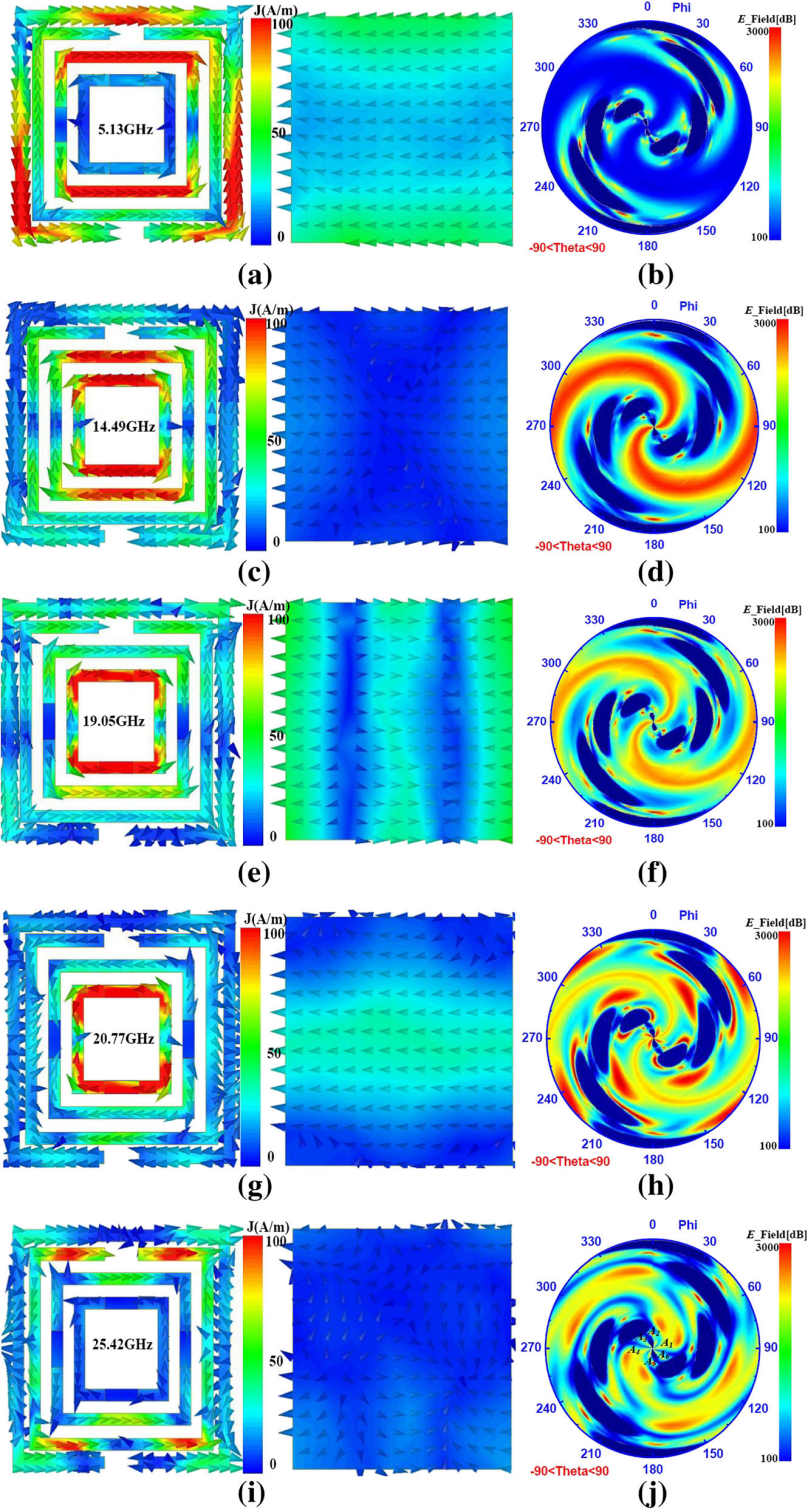
The surface current distributions of the F-MDSRR microstructure, S-MDSRR microstructure, and ground plane and the near electric fields of the PMA at the resonance frequency of 5.13, 14.49, 19.05, 20.77, and 25.42 GHz. **a** Surface current distributions at 5.13 GHz. **b** Near electric fields distributions at 5.13 GHz. **c** Surface current distributions at 14.49. **d** Near electric fields distributions at 14.49 GHz. **e** Surface current distributions at 19.05 GHz. **f** Near electric fields distributions at 19.05 GHz. **g** Surface current distributions at 20.77 GHz. **h** Near electric fields distributions at 20.77 GHz. **i** Surface current distributions at 25.42 GHz. **j** Near electric fields distributions at 25.42 GHz

To explore the mechanism for ultra-broadband absorption, the surface current distributions and the near electric fields distributions of the PMA had been given in Fig. 4 at the resonance frequency of 5.1, 14.5, 19.1, 20.8, and 25.4GHz. The exquisite resonance absorbing effect in Fig. 4a had been exhibited which were primary attributed to the SRR-I for S-MDSRR microstructure and the outer split rings for F-MDSRR microstructure at 5.13 GHz. The strong coupling between the S-MDSRR and F-MDSRR microstructures leaded to the resonance absorption. From Fig. 4c, it could be seen that the absorption peak at 14.49 GHz for proposed absorber would be obtained due to the F-MDSRR microstructure with four lumped resistances and the strong coupling in the F-MDSRR microstructure. As given in Fig. 4e, the present ultra-broadband PMA achieved absorption resonance resulting from the inter split rings for F-MDSRR and the coupling effects between the SRR-II and SRR-I. At 20.77 GHz, the absorption peak was mainly caused by the inter split rings for F-MDSRR in Fig. 4g. The strong coupling effects between the outer split rings for F-MDSRR and the SRR-II for S-MDSRR microstructure had been achieved from Fig. 4i. It was necessary to note that the dipole resonance, the equivalent inductance and capacitance resonance, and the coupling resonance were of primary importance for achieving the ultra-broadband absorption. From Fig. 4b, d, f, h, and j, it could be found that the near electric fields of 5.13 GHz in the upper space were different from that of other response frequency due to the stronger coupling effects between the SRR-I and the outer split rings. The type of the resonance absorption at 14.49, 19.1, and 20.8 GHz were same with each other, and their absorption peaks were both achieved by the F-MDSRR microstructure. It can be found that the more density of the PMA exhibited, the better absorption of the PMA achieved. As shown in Fig. 4j, there were six space points (*A*_1_, *A*_2_, *A*_3_, *A*_4_, *A*_5_, *A*_6_,) near to the origin point with strong density. These physical phenomena were all illustrated by the coupling effects and high-order modes for the proposed ultra-broadband PMA. Consequently, the coupling effects between the different microstructures and the high-order modes were the crucial component to design the broadband PMA.

**Fig. 5:**
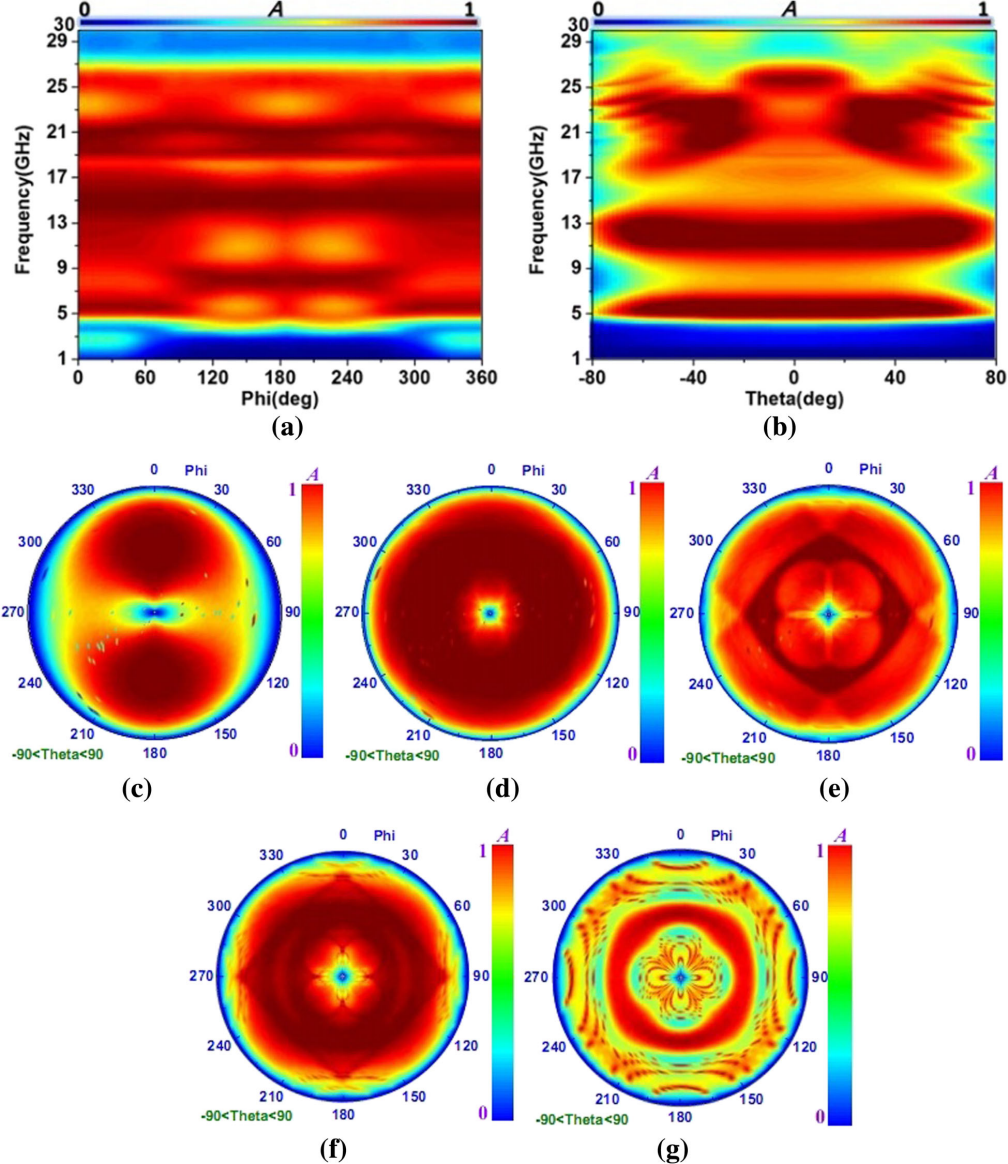
The absorption results of the present ultra-broadband PMA with different angles of theta and phi. **a** Absorption results of the PMA with different angles of phi from 1 to 30 GHz (theta = 0 deg). **b** Absorption results of the PMA with different angles of theta from 1 to 30 GHz (phi = 0°). **c** Absorption results at 5.13 GHz with − 90° < theta< 90° and 0° < phi< 360°. **d** Absorption results at 14.49 GHz with − 90° < theta< 90° and 0° < phi< 360°. **e** Absorption results at 19.05 GHz with − 90° < theta< 90° and 0° < phi< 360°. **f** Absorption results at 20.77 GHz with − 90° < theta< 90° and 0° < phi< 360°. **g** Absorption results at 25.42GHz with − 90° < theta < 90° and 0° < phi< 360°

The simulated absorption results of the present PMA with different angles of theta and phi are discussed in Fig. 5 for the transverse electromagnetic (TEM) incident waves. From Fig. 5a, we could see that the proposed PMA exhibited the high absorptivity from 4.5 to 25 GHz with theta = 0°. as the angle of phi shifted from 0 to 360°. It was obvious that the absorption decreased drastically for the angle increased from 70 to 80° or decreased from − 70 to − 80° in Fig. 5b. Generally, the ultra-broadband and wide angle absorption could be obtained for the proposed PMA with the angle of theta shifted from − 70 to 70° and the angle of phi increased from 0 to 360°. To illustrate the excellent absorption, the simulated absorption results at the resonance frequency of 5.13, 14.49, 19.05, 20.77, and 25.42 GHz are given with − 90° < theta< 90° and 0° < phi< 360° in Fig. 5c–g. From these figures, we clearly observed that the outstanding absorption at 14.49 GHz could be obtained for the PMA with − 90° < theta< 90° and 0° < phi< 360° due to the symmetrical F-MDSRR microstructure with four lumped resistances and the strong coupling effects between the inner and outer split rings. The PMA at 19.05 GHz and 20.77 GHz respectively retained high absorbing efficiency with wide absorption in Fig. 5e, f. These phenomena were proved that their absorption peaks were all achieved by the symmetrical F-MDSRR microstructure. Because the resonance of the PMA at 5.13 GHz was determined by the unsymmetrical S-MDSRR microstructure, the absorption results at this frequency were not unsymmetrical in Fig. 5c. As shown in Fig. 5g, it was necessary to point out that the absorption at 25.42 GHz was inconstancy due to the coupling effects between the F-MDSRR and S-MDSRR microstructures and the high-order modes for the proposed ultra-broadband PMA. From these figures, we could see that the proposed PMA exhibited the wide angle absorption for the electromagnetic waves with different incident angles.

**Fig. 6:**
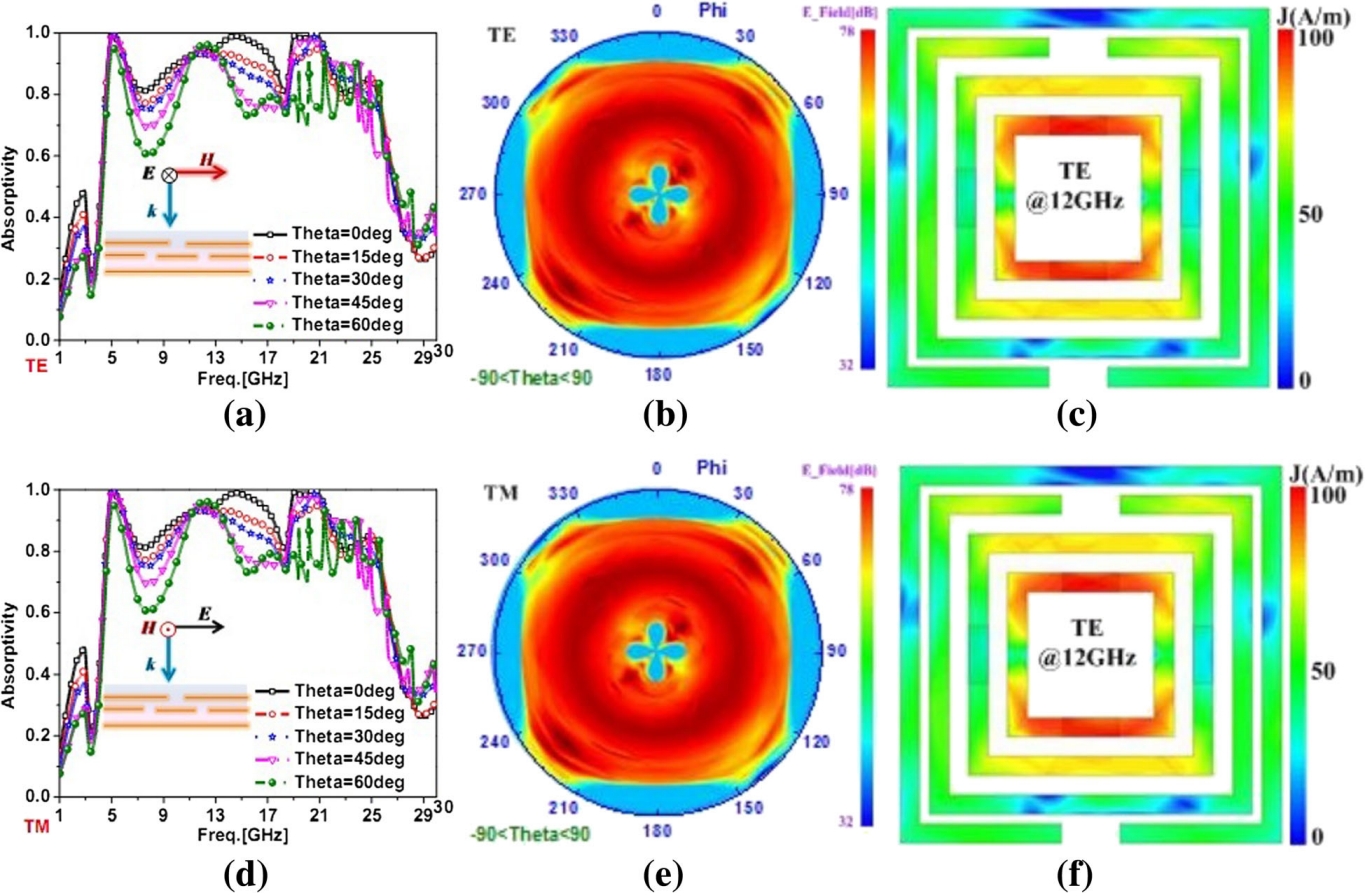
The absorption results, surface current distributions and near electric fields of the present ultra-broadband PMA with different polarized incidences. **a** The oblique absorption results of the PMA with TE polarized incidences from 1 to 30 GHz with theta shifted from 0 to 60°. **b**. The near electric fields of PMA at 12 GHz with TE polarized incidences. **c** The surface current distributions of PMA at 12 GHz with TE polarized incidences. **d** The oblique absorption results of the PMA with TM polarized incidences from 1 to 30 GHz with theta shifted from 0 to 60°. **e** The surface current distributions of PMA at 12 GHz with TM polarized incidences. **f** The near electric fields of PMA at 12 GHz with TM polarized incidences

To interpret the polarized-insensitivity of the ultra-broadband PMA for transverse electric (TE) and transverse magnetic (TM) polarized incidences, we presented the oblique absorption, the surface current distributions at 12 GHz and the near electric fields at 12 GHz in Fig. 6. From Fig. 6a, b, it is obvious that the oblique absorption results in TM polarized incidence were same with that in TE polarized incidence. The same oblique absorptions with different incidences were attributed to the absorption mechanism and the present microstructure. For example, the surface current distributions and near electric fields at 12GHz with TE and TM polarized incidences were further explored to illustrate polarized-insensitivity of the ultra-broadband PMA in Fig. 6c–f. It was reported that the presented PMA exhibited the same surface current distributions and near electric fields with different polarized incident waves. Consequently, the characteristic of polarized-insensitivity could be achieved for this ultra-broadband PMA.

**Fig. 7:**
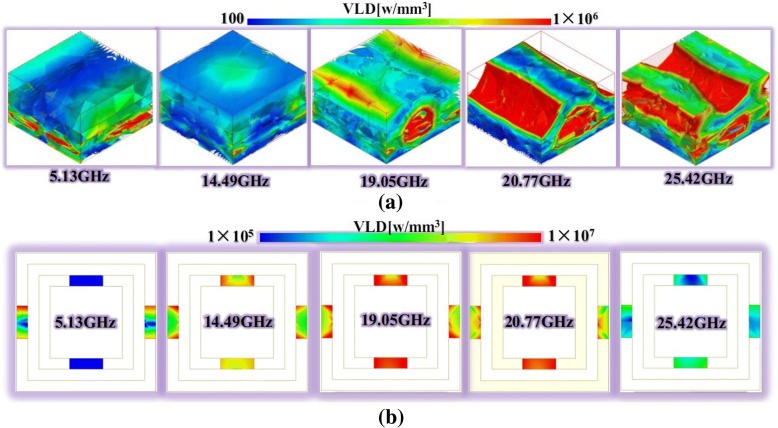
The dielectric and ohmic losses of the substrates and the lumped resistances for the proposed PAM at 5.13, 14.49, 19.05, 20.77, and 25.42 GHz. **a** The volume loss density (VLD) of substrates at the resonance frequency. **b** The volume loss density (VLD) of lumped resistances at the resonance frequency

In order to elaborate the dielectric and ohmic losses, Fig. 7 shows the volume loss density (VLD) of the substrates and lumped resistances for the proposed PAM at 5.13, 14.49, 19.05, 20.77, and 25.42 GHz. From Fig. 7a, we could observe that the VLD increased as the resonance frequency shifted from 5.13 to 25.42 GHz. The different modes could be achieved from the ohmic losses of the lumped resistances in Fig. 7b. The volume loss density of *R*_34_ was distinctly more than that of *R*_12_ at 5.13 GHz. The difference would decrease at 14.49 GHz. At 19.05 GHz and 20.77 GHz, the VLD of *R*_34_ was faintly less than that of *R*_12_. When it was 25.42 GHz, the volume loss densities of *R*_34_ and *R*_12_ were both less than that of other frequencies. It was obvious that the ohmic losses with the range from 1 × 10^5^ w/mm^3^ to 1 × 10^7^ w/mm^3^ were more than the dielectric losses with the range from 100 w/mm^3^ to 1 × 10^7^ w/mm^6^. Consequently, the ohmic and dielectric losses were important for this proposed ultra-broadband absorber from Figs. 3(e) and (f) and 7.

### Fabrication and Measurement

**Fig. 8:**
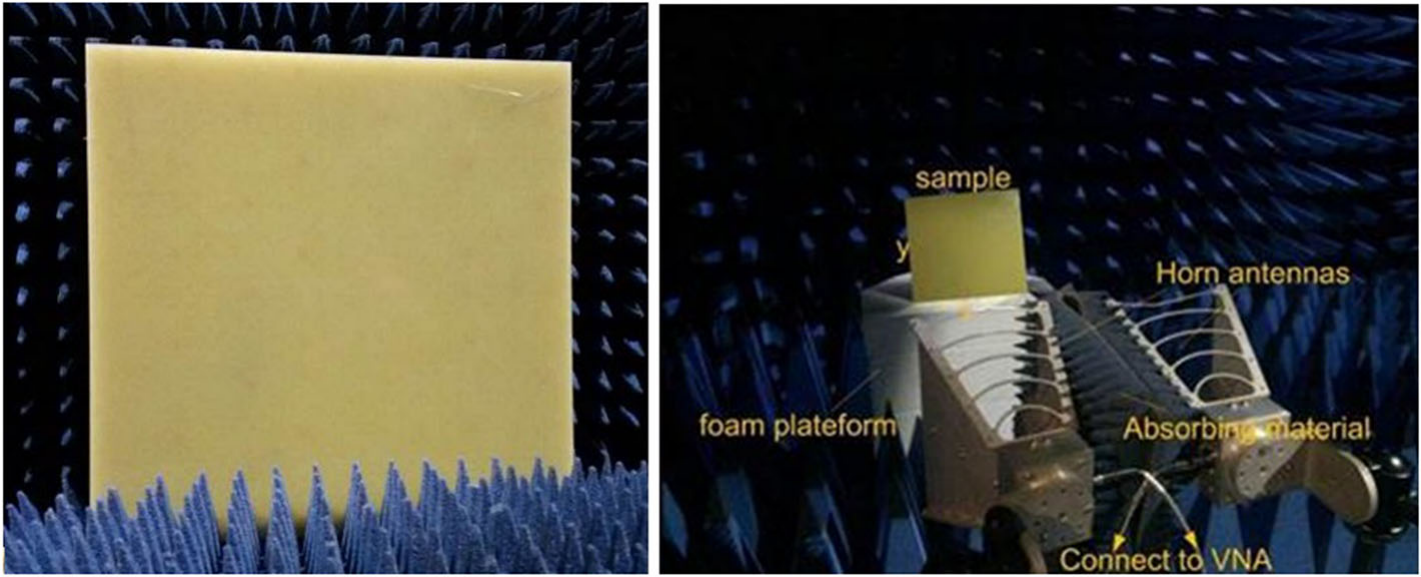
Prototypes of the proposed ultra-broadband PMA devices in a microwave anechoic chamber

In order to verify the characters, two 900-cell (30 × 30) devices of the proposed ultra-broadband PMA are fabricated and illustrated in Fig. 8. The device had been measured by employing the free-space test method in a microwave anechoic chamber. The ultra-broadband PMA sample was fabricated using an optical lithographic processes on three substrates (*ε*_r_ = 4.2 and tanδ = 0.02) with thickness of 2 mm, 1 mm, 1 mm, and 1 mm. Two linearly polarized standard-gain horn antennas as the transmitter and receiver were connected to the Agilent Vector Network Analyzer (VNA, N5230C). To eliminate the interference of environment, the function of time-domain gating in the Network Analyzer was adopted in experiments. The devices were placed vertically in the center of a turntable to ensure that the EM wave could be similar to a plane wave on the front of device. The distance between the antennas and the devices under test satisfied the far-field condition.

**Fig. 9:**
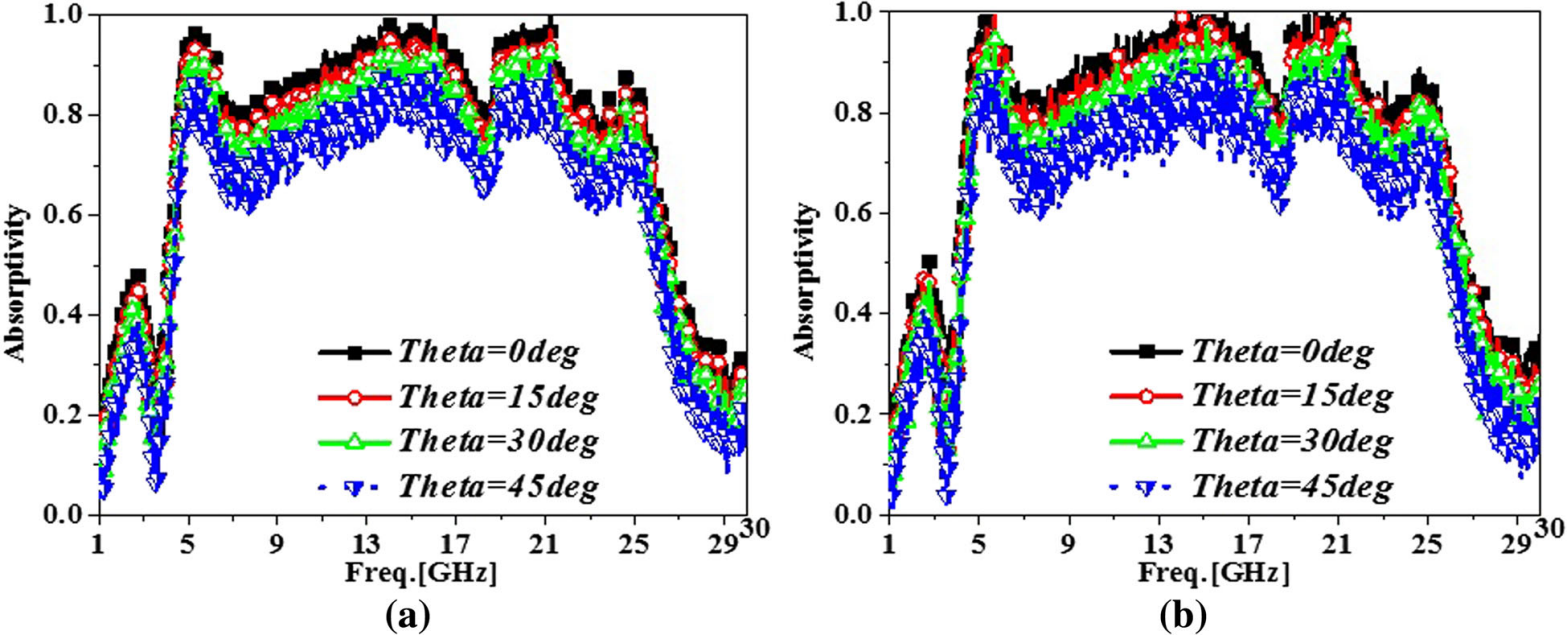
The experimental absorption for the proposed ultra-broadband PMA devices when the incident angle (*θ*) shifted from 0 to 45° in the *x*-polarized and *y*-polarized incidences. **a** The experimental absorption results of the PMA sample with *θ* of 0°, 15°, 30°, and 45° in the *x*-polarized incident waves. **b** The experimental absorption results of the PMA sample with *θ* of 0°, 15°, 30° and 45° in the *y*-polarized incident waves

The experimental results of angular absorption for the proposed PMA sample are given in Fig. 9 when the incident angle (*θ*) shifted from 0 to 45°. The measured results illustrated that the angular absorption decreased sluggishly as the incident angle increased from 0 to 45° in the *x*- and *y*- polarized incidences. When the incident angle was zero (*θ* = 0), the ultra-broadband absorption from 4.48 to 25.46 GHz could be achieved with absorptivity larger than 80% not only in *x*-polarized incidence but also in *y*-polarized incidence. Moreover, when the incident angle was 45°, the relative bandwidth of 136%, from 4.76 to 25.03 GHz, would be obtained with absorptivity larger than 60% for *x*- and *y*-polarized incident waves. From Fig. 9a, b, it was obvious that the absorptions in *x*-polarized incidences were same with that in *x*-polarized incident waves. Hence, the characteristic of polarized-insensitivity were exhibited for the proposed PMA. It was necessary to note that the absorption would exacerbate for the oblique incidence, especially with the incident angle of 45°. To improve angular absorption, the stereometamaterial structure and the substrate integrated cavity could be the beneficial candidate [22, 35]. Compared with Figs. 2(b), 6 and 9, it was clear that the experimental results agreed well with the simulated results and the presented PMA exhibited the ultra-broadband, polarized-insensitivity, and wide-incident absorption.

## Conclusion

In conclusion, we have proposed, designed, and fabricated an ultra-wideband perfect metamaterial absorber with polarized-insensitivity and wide-incident absorption. The angular absorption spectrum, surface current, and near electric-field distributions were explored to validate the excellent characteristics of the proposed perfect metamaterial absorber with strong coupling effects. The fabricated metamaterial absorber device was fabricated, measured, and analyzed. The experimental results indicated that the ultra-broadband absorption from 4.48 to 25.46 GHz could be achieved with absorptivity larger than 80% with normal incidences for *x*-polarization and *y*-polarization. For the oblique incidences with the incident angle of 45°, the perfect metamaterial absorber exhibited the relative bandwidth of 136% with absorptivity larger than 60% for different polarized incidences. This perfect metamaterial absorber device with the innovation is promising for many practical applications such as radar cross scatter reduction and electromagnetic protection in different flight platform.

## Abbreviations

EM: Electromagnetic
MDSRR: Metallic double split ring resonators
PBCs: Periodic boundary conditions
PMA: Perfect metamaterial absorber
SRR-I: Split ring resonator-I
SRR-II: Split ring resonator-II
TE: Transverse electric
TEM: Transverse electromagnetic
TM: Transverse magnetic
